# A Genetic Screen for Human Genes Suppressing FUS Induced Toxicity in Yeast

**DOI:** 10.1534/g3.120.401164

**Published:** 2020-04-10

**Authors:** Elliott Hayden, Shuzhen Chen, Abagail Chumley, Chenyi Xia, Quan Zhong, Shulin Ju

**Affiliations:** * Department of Biological Sciences, Wright State University, Dayton, OH 45435 and; ^†^ School of Basic Medicine, Shanghai University of Traditional Medicine, Shanghai, China 201203

**Keywords:** FUS, ALS, human gene suppressors, yeast, genetic screen

## Abstract

FUS is a nucleic acid binding protein that, when mutated, cause a subset of familial amyotrophic lateral sclerosis (ALS). Expression of FUS in yeast recapitulates several pathological features of the disease-causing mutant proteins, including nuclear to cytoplasmic translocation, formation of cytoplasmic inclusions, and cytotoxicity. Genetic screens using the yeast model of FUS have identified yeast genes and their corresponding human homologs suppressing FUS induced toxicity in yeast, neurons and animal models. To expand the search for human suppressor genes of FUS induced toxicity, we carried out a genome-scale genetic screen using a newly constructed library containing 13570 human genes cloned in an inducible yeast-expression vector. Through multiple rounds of verification, we found 37 human genes that, when overexpressed, suppress FUS induced toxicity in yeast. Human genes with DNA or RNA binding functions are overrepresented among the identified suppressor genes, supporting that perturbations of RNA metabolism is a key underlying mechanism of FUS toxicity.

ALS is a neurodegenerative disease characterized by the degeneration of upper and lower motor neurons in the brain and spinal cord leading to progressive paralysis and ultimately death within five years of symptom onset. FUS is a multifunctional RNA-binding protein involved in diverse RNA metabolic processes. Mutations in FUS cause an inherited form of ALS (Kwiatkowski *et al.* 2009). Several features associated with FUS pathology, including formation of cytoplasmic inclusions and cytotoxicity, have been recapitulated in the budding yeast *Saccharomyces cerevisiae*, enabling us and others to employ this powerful genetic system to rapidly identify genetic suppressors (Ju *et al.* 2011; Sun *et al.* 2011b). A critical resource used in such genetic screens is a library consisting of ∼5,000 sequence verified protein-coding yeast genes (Hu *et al.* 2007). Screening against this library has identified yeast genes that, when overexpressed, reduce the toxicity of FUS (Ju *et al.* 2011; Sun *et al.* 2011b). Consistent with the existence of conserved cellular mechanisms underlying FUS induced toxicity from yeast to human, UPF1, the human homolog of a yeast suppressor gene, shows strong protective effect in yeast, neurons and animal models of ALS (Barmada *et al.* 2015; Jackson *et al.* 2015; Xu *et al.* 2019). UPF1 is an essential component of the nonsense mediated mRNA decay (NMD) pathway, which detects and directs mRNAs with premature stop codons for degradation thus preventing the accumulation of truncated proteins (He and Jacobson 2015). NMD plays a critical role in mRNA surveillance, and is conserved from yeast to human (He and Jacobson 2015). Convergent suppressor mechanisms of FUS induced toxicity in yeast and mammalian systems supports the use of yeast as a model to study FUS toxicity.

We reasoned that direct expression of human genes in yeast may expand the search for genetic modifiers of cytotoxicity induced by disease-associated proteins. The obstacle to set up such a genetic screen is the availability of the full collection of human protein-coding genes cloned in a yeast expression vector. With Gateway cloning technology and a collection of 13570 human genes as entry clones generously provided to us by Dr. Marc Vidal, we generated a library of human genes individually cloned on a yeast expression vector, pAG416GAL-ccdB (Alberti *et al.* 2007). Using this library, we screened for human suppressor genes of toxicity mediated by ALS-associated protein FUS.

Overexpression genetic screen is typically done by individually transforming a library of plasmid clones into a yeast model of interest (Willingham *et al.* 2003; Cooper *et al.* 2006; Ju *et al.* 2011; Sun *et al.* 2011b). We developed a more efficient and cost-effective method for the screen that relies on highly efficient yeast mating rather than transformation (Hayden *et al.* 2018). Using this method, we introduced the arrayed library of human genes into the yeast model of FUS and screened for human suppressor genes. After several round of verifications, we confirmed 37 human genes that, when overexpressed, strongly reduce FUS induced toxicity. Gene ontology (GO) enrichment analysis revealed that FUS suppressors are enriched in genes with nucleic acid or RNA binding functions. Given that our lab and others have previously identified FUS suppressors genes involved in RNA metabolism (Ju *et al.* 2011; Sun *et al.* 2011b), expression of the suppressor human RNA-binding proteins in yeast may counter deleterious effects of FUS on RNA processing, transport or stability.

## Materials and Mehtods

### Human ORF clones

The human ORF clones used to generate the yeast-expression plasmids are Gateway entry clones kindly provided by Dr. Marc Vidal (Center for Cancer Systems Biology at Dana-Farber Cancer Institute and Department of Genetics, Harvard Medical School). The collection of human ORF clones, including the clones from hORFeome version 8.1 (Yang *et al.* 2011)(http://horfdb.dfci.harvard.edu/) and clones from the ORFeome collaboration (Collaboration 2016)(http://www.orfeomecollaboration.org), have been sequence verified previously. All ORF clones were cloned into the pAG416GAL-ccdB vector (Alberti *et al.* 2007) using Gateway LR recombination cloning (Hartley *et al.* 2000; Walhout *et al.* 2000). The resulting expression clones of human genes were subsequently transformed into the haploid w303 yeast strain (*MAT*α *can1**-100*, *leu2**-3,112*, *trp1**-1*, *ura3**-1*, *ade2**-1*, *his3**-11,15*) using a high-throughput yeast transformation protocol as previously described (Fleming and Gitler 2011; Hayden *et al.* 2018). Transformed yeast strains bearing the expression plasmids of human genes were arrayed in 96-well plates and stored as glycerol archives at -80°.

### Yeast strains and growth media

The 1xFUS integration strain was generated in the haploid W303 yeast strain (*MAT*a *can1**-100*, *leu2**-3,112*, *trp1**-1*, *ura3**-1*, *ade2**-1*, *his3**-11,15*::pRS303Gal1FUS) as previously described (Ju *et al.* 2011). YPD media (1% yeast extract, 2% peptone and 2% glucose in distilled water) was used for mating. Synthetic dropout media lacking uracil (Ura-) was used for growing the haploid yeast strains containing the expression plasmids of human genes. Synthetic dropout media lacking both uracil and histidine (Ura- His-) was used for growing the diploid yeast strains containing both FUS integration and the human-gene plasmids. Yeast media contained 2% glucose, galactose or raffinose as the carbon source.

### Genetic screen and verification

Detailed methods of screening by mating have been previously described (Hayden *et al.* 2018). Briefly, the haploid 1xFUS integration strain was crossed individually with each of the archived haploid yeast strains containing the expression clones of the human genes. The resulted diploid yeast strains were selected in Ura- His- liquid media, and plated onto Ura- His- agar plates containing glucose and galactose respectively, using the Singer RotoR robotic equipment (Singer Instruments). Following incubation at 30°, pictures of yeast colonies grown on the agar plates were taken daily until day four. Suppressors of FUS were identified by visually inspecting galactose agar plates. Yeast strains that grew clearly better than the background growth of the diploid FUS model on galactose plates were considered as screening hits. The corresponding human gene plasmids of all screening hits were isolated, transformed in the haploid w303 *MAT*α yeast strain. Each transformants was crossed with the haploid 1xFUS integration strain. The resulting diploid strains were then grown to mid-log phase in Ura- His- liquid media containing Raffinose. Next, cell cultures were normalized to OD_600_ at 1.0, serially diluted by five folds and spotted onto the respective Ura- His- dropout agar plates containing 2% glucose and galactose respectively. Agar plates were incubated at 30° for three days and images of yeast colonies were taken. The identity of each verified human suppressor gene was confirmed by Sanger end-read sequencing and alignment to the expected sequence of the ORF clone.

### Go term enrichment analysis

Go term enrichment analysis was conducted using PANTHER overrepresentation test. The reference gene list used to run the analysis included the 13570 Entrez gene IDs corresponding to all the cloned human ORF clones in the library. The annotation datasets used for the analysis was GO molecular function, biological process and cellular component released on 12/09/2019. Statistical analyses include Fisher’s Exact Test followed by FDR correction. All GO terms with a fold enrichment greater than or equal to three and FDR of less than or equal to 0.05 were included in Table 2.

### Data availability

Strains and plasmids are available upon request. The authors affirm that all data necessary for confirming the conclusions of the article are present within the article, figures, and tables.

## Results and Discussion

### Identification of human gene suppressing the toxicity of FUS in yeast

With the ease to carry out genome-wide genetic screens, yeast has served as a model for studying many human disease-associated proteins. One of such genetic screens, involving systematic overexpression of yeast genes, was used to screen yeast suppressor genes of FUS toxicity. Dosage-dependent genetic rescue of FUS toxicity has also been confirmed for the human homolog of a yeast suppressor gene, leading to the identification of conserved mechanisms underlying FUS pathology (Ju *et al.* 2011; Barmada *et al.* 2015; Jackson *et al.* 2015).

**Figure 1:**
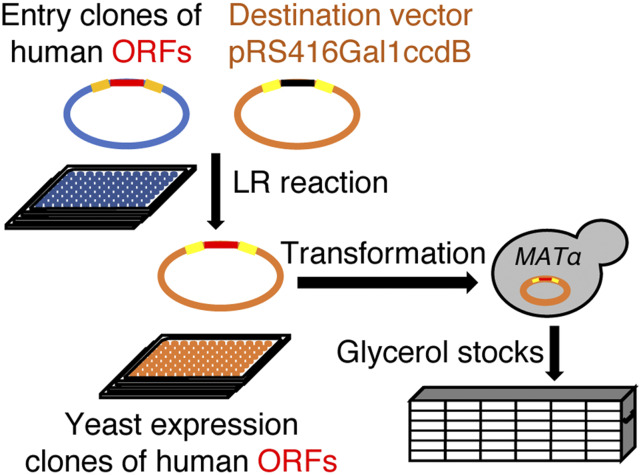
Generation of yeast-expression human-gene library. The human ORFs were cloned into the yeast expression vector, pRS416Gal1ccdB, by Gateway LR cloning. The yeast-expression plasmids containing the human ORFs (pRS416Gal1hORFs) were isolated and transformed into W303 *MAT*α strain. Yeast strains, each containing an unique human ORF, were arrayed in 96-well microplates, and stored as a glycerol stock at -80 ° for later use.

More than 50% of human genes, however, do not have readily detectable homologs in yeast. Human genes without yeast homologs cannot be identified using the current genetic screens in yeast. A lack of sequence similarity does not necessarily exclude the possibility of such non-conserved human genes to function in yeast. Non-conserved human proteins may interact directly with FUS, suppressing its cytotoxicity in yeast. Alternatively, non-conserved human proteins may form inter-species protein-protein interactions with yeast proteins (Zhong *et al.* 2016), indirectly modulating the toxicity of FUS. To expand the search for new human gene targets related to FUS toxicity in yeast, we developed a new genomic tool that allows systematic overexpression of human genes in yeast (Figure 1). To do so, we first obtained a collection of sequence verified human ORFs as Gateway entry clones (corresponding to 13570 genes) (Yang *et al.* 2011; Collaboration 2016). While the collection does not contain all human genes, it was developed through unbiased large-scale cloning efforts and represents a key resource for systematic unbiased functional studies of human genes. We individually amplified the plasmid DNA of each entry clone and carried out Gateway LR reaction to transfer each human ORF into a yeast expression vector, pRS416Gal1ccdB (Alberti *et al.* 2007). The human ORFs cloned in the pRS416Gal1ccdB vector are under the control of the *GAL1* promoter, which is induced upon shifting to galactose containing media.

**Figure 2:**
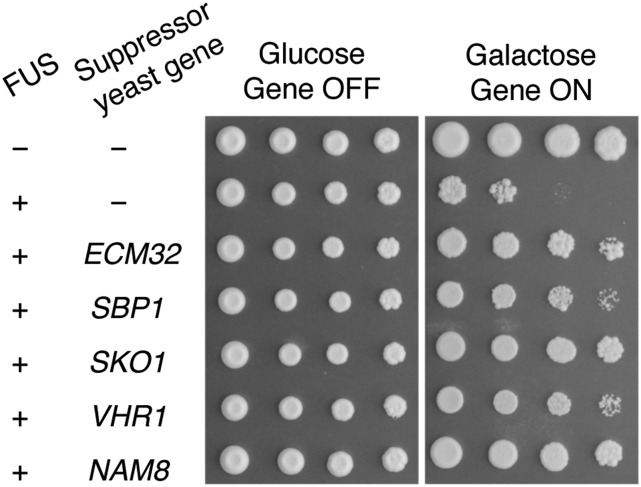
Previously identified yeast suppressor genes rescue FUS toxicity in diploid yeast. Plasmids containing previously identified five yeast genes that suppress FUS toxicity were transformed into a haploid yeast strain w303 *MAT*α. Transformed yeast were crossed with the FUS model generated in the isogenic yeast strain with the opposite mating type, W303 *MAT*a. Diploid yeast were selected, serially diluted, and spotted onto agar plates containing glucose (genes-off condition) and galactose (genes-on condition), respectively. Row 1 shows a control yeast strain containing two empty vectors. Row 2 shows the diploid FUS yeast strain with an empty vector, where expression of FUS is toxic. Rows 3-7 show diploid yeast expressing FUS as well as a suppression gene that can rescue FUS toxicity. The picture, representing three independent experiments, was taken after growth at 30° for three days.

Previously, high-throughput yeast transformation was used to introduce the plasmids bearing the gene to be overexpressed. Preparation of the library of plasmids can be time consuming and costly. Considering that the human-gene library contains many more clones than the yeast-gene libraries, we tested a method that relies on highly efficient yeast mating instead of transformation to introduce the arrayed plasmid library of human ORF clones into the yeast model (Hayden *et al.* 2018). First, we verified that yeast suppressor genes previously identified in the haploid yeast model of FUS (Ju *et al.* 2011) also exhibited suppressor effects on FUS toxicity in diploid yeast (Figure 2). This result indicates that screening for suppressors of FUS toxicity may be carried out in a diploid strain background. Next, we introduced the human-gene library into the W303 *MAT*α strain, the isogenic but opposite mating type of the haploid yeast model of FUS (Ju *et al.* 2011). We arrayed yeast transformants, each with one human-gene expression clone in microplates and archived them as glycerol stock.

**Figure 3:**
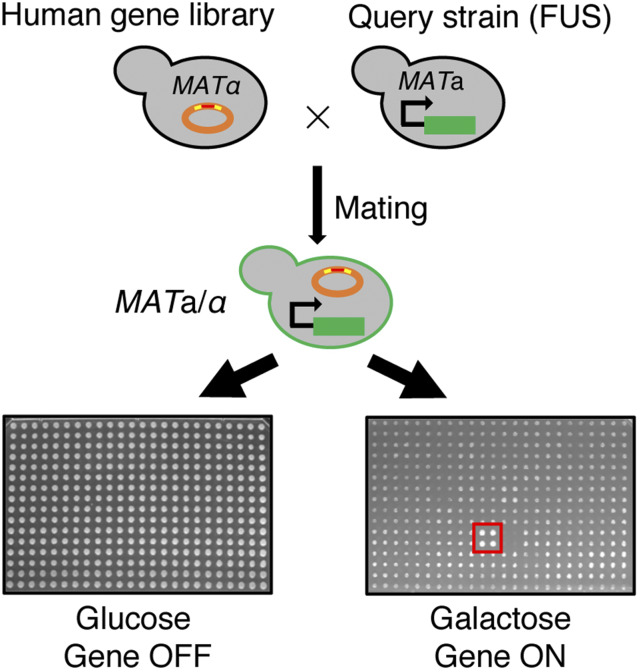
A mating-based strategy to screen for human suppressor genes rescuing FUS toxicity upon overexpression. W303 *MAT*α yeast containing the expression clones of human ORFs were revived from the glycerol stock and crossed with the FUS model generated in the isogenic yeast strain with the opposite mating type, W303 *MAT*a. Diploid yeast strains were selected and spotted in quadruplicate, using the Singer RotoR robotic equipment, onto agar plates containing glucose (genes-off condition) and galactose (genes-on condition), respectively. On the galactose plates, most yeast had severely reduced growth due to the expression of FUS. The red square labels a screening hit of a human ORF clone that suppresses FUS toxicity allowing yeast to form much larger colonies.

For the screen, we revived the arrayed human-gene library strains and individually crossed to the yeast model of FUS in YEPD. The resulting diploid yeast strains containing both the integrated FUS gene and the human genes from the library were selected. Diploid yeast cells were robotically pinned in quadruplicate onto agar plates containing either glucose or galactose as the carbon source. Since FUS expression is highly toxic to yeast, growth for most diploid strains was reduced on galactose as compare to glucose. In contrast, the growth for a subset of the strains was better, indicating the suppression of FUS toxicity by simultaneously expression of another human gene (Figure 3).

**Figure 4:**
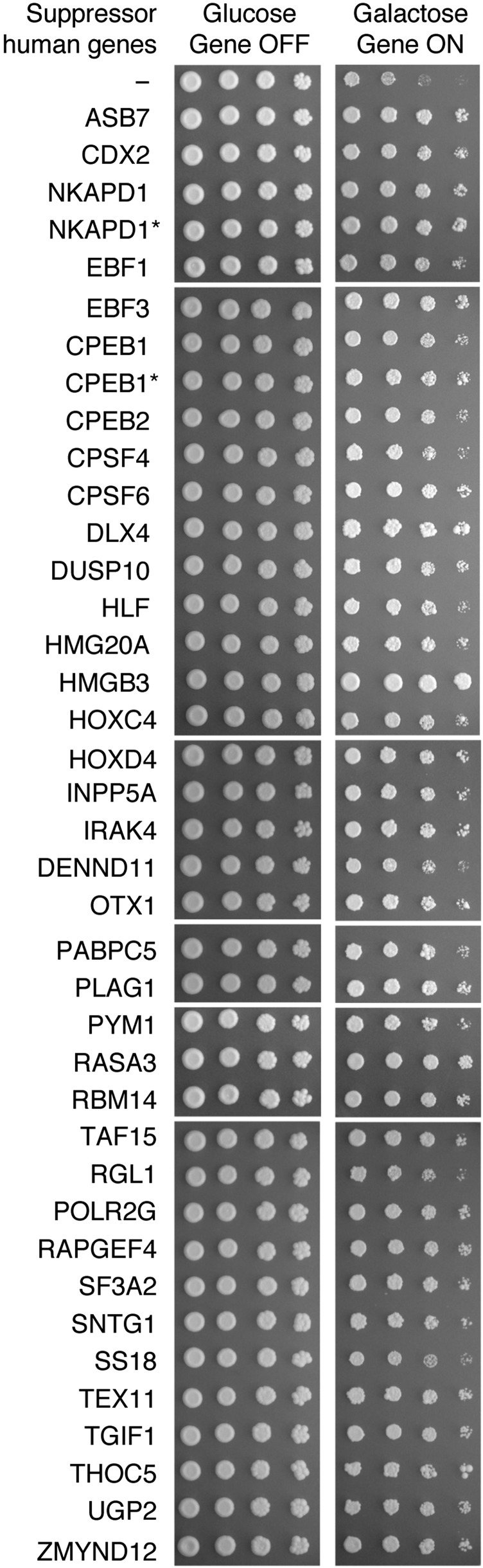
Human suppressor genes rescue FUS toxicity upon overexpression. W303 *MAT*α containing the empty vector, pRS416Gal1ccdB, or each of the identified human gene that suppress FUS toxicity were crossed with the FUS model generated in the isogenic strain with the opposite mating type, W303 *MAT*a. Diploid yeast strains were selected, serially diluted and spotted onto agar plates containing glucose (genes-off condition) and galactose (genes-on condition), respectively. The picture, representing three independent experiments, was taken after growth at 30° for three days. *indicating different ORF clones of the same gene.

After screening the entire library, we cherrypicked the expression clones of all screening hits and re-transformed them into w303 *MAT*α. We retested the suppressor effect of all new transformants using the mating method. Finally, we confirmed their suppressor phenotype by serial dilution spotting assay (Figure 4). In total, we identified 37 human ORF clones corresponding to 37 human genes (Table 1), that suppress FUS toxicity when overexpressed.

**Table 1:** List of human genes that suppress FUS toxicity

Gene Name	Short Description
ASB7	Belongs to a family of ankyrin repeat proteins that regulate protein turnover by targeting proteins for polyubiquitin and proteasome-mediated degradation
CDX2	Member of caudal-related homeobox transcription factor family
CPEB1	Binding to the polyadenylation element, and regulating mRNA translation and processing
CPEB2	Regulating translation of target mRNAs by binding to the polyadenylation element in the 3′UTR
CPSF4	Component of the cleavage and polyadenylation specificity factor complex
CPSF6	Subunit of a cleavage factor required for 3′ RNA cleavage and polyadenylation processing
DENND11	Probable guanine nucleotide exchange factor (GEF)
DLX4	Member of Distal-less family of homeobox-containing genes
DUSP10	Protein phosphatase involved in the inactivation of MAP kinases
EBF1	Transcription factor expressed in early B-cells, adipocytes, and olfactory neurons.
EBF3	Member of the transcription factors that are involved in B-cell differentiation, bone development and neurogenesis
HLF	Homodimers or heterodimers with other PAR family members to activate transcription
HMG20A	Chromatin-associated protein playing a role in neuronal differentiation
HMGB3	Associates with chromatin and binds DNA with a preference to non-canonical DNA structures
HOXC4	Member of homeobox family of genes that play important roles in morphogenesis
HOXD4	Member of homeobox family of genes that play important roles in morphogenesis
INPP5A	Membrane-associated type I inositol 1,4,5-trisphosphate 5-phosphatase
IRAK4	Serine/threonine-protein kinase that plays a role in initiating innate immune response against foreign pathogens. Involved in Toll-like receptor (TLR) and IL-1R signaling pathways
NKAPD1	Uncharacterized protein C11orf57
OTX1	Homeodomain-containing transcription factor, playing a role in brain development
PABPC5	Binding to the polyA tail and playing a role in mRNA metabolic processes in the cytoplasm
PLAG1	Proto- oncogene, belongs to the PLAG family of zinc finger transcription factors
POLR2G	Subunit of RNA polymerase II, the polymerase responsible for synthesizing mRNA
PYM1	Regulator of the exon junction complex (EJC) as an EJC disassembly factor
RAPGEF4	Guanine nucleotide exchange factor that is activated by binding cAMP
RASA3	Inhibitory regulator of the Ras-c AMP pathway
RBM14	Ribonucleoprotein functioning as a general nuclear coactivator, and an RNA splicing modulator
RGL1	Probable guanine nucleotide exchange factor
SF3A2	A component of the splicing factor SF3A complex involved in pre-mRNA splicing
SNTG1	Member of the syntrophin family, involved in mediating gamma-enolase trafficking to the plasma membrane and in enhancing its neurotrophic activity
SS18	Component of SWI/SNF chromatin remodeling subcomplex
TAF15	Member of the TET family of RNA-binding proteins
TEX11	Regulator of crossing-over during meiosis
TGIF1	Conserved transcription regulator which binds to retinoid X receptor responsive element
THOC5	Component the TREX complex which specifically associates with spliced mRNA, and which is thought to couple mRNA transcription, processing and nuclear export
UGP2	Essential enzyme that catalyzes the conversion of glucose-1- phosphate to UDP-glucose
ZMYND12	Zinc finger MYND domain-containing protein 12

### Human suppressor genes of FUS toxicity

The identified suppressor genes appear to have a wide range of functions. GO enrichment analysis identified several functional groups of genes are overrepresented (Table 2), particularly those with RNA or DNA binding functions. FUS is a nucleic acid binding protein that has been implicated in transcription regulation, RNA splicing, RNA transport, and RNA stability (Polymenidou *et al.* 2012; Butti and Patten 2018). The identification of the group of suppressor genes with similar functions supports the idea that expression of FUS perturbs RNA metabolism in yeast, thus inducing cellular toxicity.

**Table 2:** Enriched functions of the human-gene suppressors of FUS toxicity

GO terms	GO ID	GO category	Enrichment folds	*P* value	FDR
mRNA 3′-UTR binding	GO:0003730	Molecular function	15.8	1.43E-04	4.92E-02
mRNA binding	GO:0003729	Molecular function	10.6	1.01E-06	2.25E-03
DNA-binding transcription activator activity, RNA polymerase II-specific	GO:0001228	Molecular function	6.3	4.05E-05	3.03E-02
DNA-binding transcription activator activity	GO:0001216	Molecular function	6.2	4.30E-05	2.75E-02
RNA polymerase II regulatory region sequence-specific DNA binding	GO:0000977	Molecular function	4.4	8.05E-05	4.51E-02
RNA polymerase II regulatory region DNA binding	GO:0001012	Molecular function	4.4	8.16E-05	4.06E-02
Transcription regulatory region sequence-specific DNA binding	GO:0000976	Molecular function	4.1	1.31E-04	4.87E-02
Double-stranded DNA binding	GO:0003690	Molecular function	3.9	9.27E-05	4.15E-02
DNA-binding transcription factor activity, RNA polymerase II-specific	GO:0000981	Molecular function	3.9	1.00E-04	4.09E-02
DNA-binding transcription factor activity	GO:0003700	Molecular function	3.4	1.65E-04	4.61E-02
Transcription regulator activity	GO:0140110	Molecular function	3.2	3.60E-05	3.23E-02
RNA binding	GO:0003723	Molecular function	3.1	5.85E-05	3.73E-02
Embryonic organ development	GO:0048568	Biological process	6.6	2.80E-05	2.03E-02
Positive regulation of RNA metabolic process	GO:0051254	Biological process	3.7	6.31E-07	4.81E-03
Positive regulation of transcription, DNA-templated	GO:0045893	Biological process	3.7	3.73E-06	5.68E-03
Positive regulation of macromolecule biosynthetic process	GO:0010557	Biological process	3.5	6.52E-07	3.31E-03
positive regulation of nucleic acid-templated transcription	GO:1903508	Biological process	3.4	7.93E-06	9.30E-03
Positive regulation of RNA biosynthetic process	GO:1902680	Biological process	3.4	8.01E-06	8.72E-03
Positive regulation of nucleobase-containing compound metabolic process	GO:0045935	Biological process	3.4	2.37E-06	4.02E-03
Positive regulation of cellular biosynthetic process	GO:0031328	Biological process	3.3	1.39E-06	4.23E-03
Positive regulation of gene expression	GO:0010628	Biological process	3.3	1.39E-06	3.53E-03
Positive regulation of biosynthetic process	GO:0009891	Biological process	3.3	1.78E-06	3.87E-03
Nuclear chromatin	GO:0000790	Cellular component	4.2	2.01E-05	1.29E-02
Nuclear chromosome	GO:0000228	Cellular component	4.0	6.54E-06	1.26E-02
Chromatin	GO:0000785	Cellular component	3.6	9.32E-05	4.50E-02

FDR correction < 0.05; Fold of enrichment > 3.

### RNA binding or splicing

Cellular toxicity of FUS has been speculated to involve binding and sequestering of essential proteins and mRNAs into persistent intracellular inclusion bodies (Ciryam *et al.* 2017; Hayden *et al.* 2017). Consistent with this idea, previous studies using a yeast model of FUS have shown that FUS aggregation colocalizes with stress granules(Sun *et al.* 2011b) The identified human-gene suppressors in this group may compete to bind yeast mRNAs and protect them from being sequestered into FUS inclusions.

CPEB1 and CPEB2: Cytoplasmic polyadenylation element-binding protein 1 and 2 are member of the cytoplasmic polyadenylation element binding protein family, which promotes polyadenylation induced translation. CPEB proteins are involved in a wide range of cellular functions, including germ-cell development, cell division, synaptic plasticity, learning and memory (Richter 2007; Ivshina *et al.* 2014).

CPSF4 and CPSF6: Cleavage and polyadenylation specificity factor subunit 4 and 6 are essential components of the 3′ end processing machinery of pre-mRNAs. The cleavage factor complex plays a key role in pre-mRNAs 3′-cleavage and polyadenylation. Studies have found overexpression of CPSF4 in several types of cancer, such as lung, breast, and colorectal cancer (Zhang *et al.* 2016; Wu *et al.* 2019). Mutation studies indicate CPSF6 functions in regulating poly A site selection and preventing premature 3′-UTR cleavage (Sasado *et al.* 2017). It was reported that the interaction of CPSF6 with HIV-1 capsid plays a critical role for targeting integration of HIV-1 to transcriptionally active chromatin (Sowd *et al.* 2016). CPSF6 is also implicated in breast cancer as a tumor promoting factor (Binothman *et al.* 2017).

PABPC5: Poly(A)-binding protein 5 is a protein that binds to the polyA tail of eukaryotic mRNAs. It plays a critical role in the regulation of mRNA transport and mRNA decay in the cytoplasm (Bhattacharjee and Bag 2012).

POLR2G: RNA polymerase II subunit B7 is a conserved protein that is part of the large 12-subunit RNA polymerase II. Together with another subunit B4, POLR2G not only is important for initiation of transcription, but also plays critical roles in diverse cellular processes, such as mRNA export, mRNA decay, DNA repair, protein translation, and stress response (Sharma and Kumari 2013; Kumar *et al.* 2019).

PYM1: Partner of Y14 and mago (PYM homolog 1) is an exon junction complex-associated factor that play an important role in exon-exon junction complex disassembly. It is also involved in the nonsense-mediated decay pathway and regulation of protein translation (Diem *et al.* 2007).

RBM14: RNA-binding motif protein 14 is an RNA binding protein that is involved in multiple important cellular processes, such as transcriptional regulation (Li *et al.* 2017), DNA damage response (Yuan *et al.* 2014; Simon *et al.* 2017), genome integrity maintenance (Shiratsuchi *et al.* 2015; Li *et al.* 2020), and maintaining the pluripotency of embryonic stem cells (Chen *et al.* 2018).

SF3A2: Splicing factor 3A subunit 2 is a subunit of the splicing factor 3a protein complex that includes subunits 1, 2 and 3 and is necessary for the assembly of spliceosome that plays a critical role in pre-mRNA splicing (Bennett and Reed 1993). Together with Prp31, it also has a direct role in mitotic chromosome segregation(Pellacani *et al.* 2018).

TAF15: Mutations in TAF15 were found in familial form of ALS (Couthouis *et al.* 2011; Ticozzi *et al.* 2011). TAF15 encodes a TATA-binding protein-associated factor that functions in promoter recognition, transcription initiation complex assembly, and transcription activation (Kapeli *et al.* 2017).

THOC5: THO complex subunit 5 is a conserved protein in the THO complex that functions in processing and transport of mRNA (Scott *et al.* 2019). THOC5 is phosphorylated upon extracellular stimuli suggesting its role in DNA damage response (Ramachandran *et al.* 2011), growth factor/cytokine-mediated differentiation, and cancer development (Wang *et al.* 2013; Tran *et al.* 2016). New evidence also implicates this protein in synapse development and dopamine neuron survival (Maeder *et al.* 2018).

### DNA binding or transcription factors

Overexpression of FUS in yeast may affect the expression of some yeast genes and subsequently disrupt their cellular functions and reduce cell fitness. Although the transcription factors identified from this screen may not directly target yeast genes and regulate their expression, it is possible that the suppressor proteins interact with FUS or with other yeast transcriptional factors, thus indirectly reverse the detrimental effect of FUS on yeast gene expression.

CDX2: Homeobox protein CDX-2 is a transcription factor that plays import roles in cell differentiation and proliferation. It is a well-known cancer risk factor. Abnormal CDX2 expression was indicated in metastasis of multiple cancers such as esophagus, stomach, colon, breast, ovarian, prostate cancer, and leukemia (Chawengsaksophak 2019).

DLX4: Homeobox protein DLX-4 is a transcription factor that belongs to the Distal-less (Dlx) family of proteins. DLX4 plays important functions in cell differentiation, proliferation, and migration. Abnormal DLX4 expression is associated with multiple cancers and preeclampsia (Sun *et al.* 2011a; Zhang *et al.* 2012). In addition, it was reported that DLX4, functionally replacing c-Myc, promotes generation of human induced pluripotent stem cells (Tamaoki *et al.* 2014).

EBF1 and EBF3: both genes encode highly conserved Collier/Olf/EBF (COE) family of transcription factors. EBF1 controls the expression of key proteins in B cell differentiation, signal transduction and function (Vilagos *et al.* 2012; Kong *et al.* 2016). EBF3 is thought to have functions in the nervous system, as mutations in EBF3 cause hypotonia, ataxia, and delayed development syndrome, a genetic neurodevelopmental syndrome (Chao *et al.* 2017).

HOXC4 and HOXD4: Homeobox proteins C4 and D4 are a group of related transcription factors that play important roles in specifying regions of the body plan during development, ensuring that the structures form in the correct places (Rastegar *et al.* 2004; Nolte *et al.* 2006). Expression of HOXC4 and HOXD4 was elevated in breast cancer, myeloid leukemias and uveal melanoma, suggesting its role as a potential oncogene (Bijl *et al.* 1997; Wu *et al.* 2020).

HLF: HLF gene encode a protein called hepatic leukemia factor. A Chimeric protein between HLF and E2A resulting from the leukemogenic translocation is responsible for a rare form of acute lymphoblastic leukemia. This arises from both impairment of normal function of E2A and TCF3 as a tumor suppressor in T lymphocytes, and activation of survival pathway triggered through HLF DNA binding domain (Seidel and Look 2001; Panagopoulos *et al.* 2012).

HMGB3 and HMG20A: both genes encode members of the HMG-box superfamily of DNA-binding proteins. HMGB 3 encodes the high mobility group protein B3, which plays an important function in maintaining stem cell populations, and increased expression of HMGB3 is a risk factor for several type of cancer, possibly through its regulation on WNT/β-catenin pathway (Gao *et al.* 2015; Zhang *et al.* 2017; Lv *et al.* 2019). HMG20A encodes high mobility group protein 20A, a close homolog HMG20B that plays important roles in neuronal differentiation (Artegiani *et al.* 2010), and is required for SNAI1-mediated epithelial to mesenchymal transition (Rivero *et al.* 2015).

HOXC4 and HOXD4: Homeobox proteins C4 and D4 are a group of related transcription factors that play important roles in specifying regions of the body plan during development, ensuring that the structures form in the correct places (Rastegar *et al.* 2004; Nolte *et al.* 2006). Expression of HOXC4 and HOXD4 was elevated in breast cancer, myeloid leukemias and uveal melanoma, suggesting its role as a potential oncogene (Bijl *et al.* 1997; Wu *et al.* 2020).

OTX1: OTX1 is a member of the bicoid subfamily of homeodomain-containing transcription factors that play critical roles in brain and sensory organ development (Simeone 1998; Zhang *et al.* 2015). Abnormal expression of OTX1 is also recently reported as a risk factor for various cancers, including neuroblastoma, breast, liver, gastric and colorectal cancer(Li *et al.* 2016; Qin *et al.* 2018; Li *et al.* 2019; Micheloni *et al.* 2019; Yang *et al.* 2019).

PLAG1: Pleomorphic adenoma gene 1 (PLAG1) encodes a zinc finger transcription factor, which is developmentally regulated. Study of PLAG1 knockout mice suggests it is an important regulator in postnatal growth and reproduction (Juma *et al.* 2016). Overexpression and knockdown of PLAG1 also indicate its role in regulating neuronal gene expression and neuronal differentiation of neocortical neural progenitor cells(Sakai *et al.* 2019).

TGIF1: TGIF1 belongs to the three-amino acid loop extension (TALE) superclass of atypical homeodomain proteins that function as transcription regulators. It plays an important function in normal brain development. Mutations in this gene are associated with holoprosencephaly type 4, which is a structural anomaly of the brain (Wotton and Taniguchi 2018; Zhu *et al.* 2018). Abnormal expression of TGIF1 has also been implicated in many cancers.

### Genes involved in cell signaling

DUSP10: Dual specificity protein phosphatase 10 is an enzyme that inactivates stress-activated kinases, such as p38 and SAPK/JNK, by dephosphorylation. The gene is ubiquitously expressed, and its expression is highly induced upon stress stimuli. Studies indicated a clear role of DUSP10 in inflammation, immunity, and cancer (Jimenez-Martinez *et al.* 2019).

RAPGEF4: Rap guanine nucleotide exchange factor (GEF) 4 is a protein that is targeted by the second messenger cAMP, and functions as a guanine nucleotide exchange factor for the Ras-like small GTPase Rap upon cAMP stimulation. It is involved in various cellular processes such as integrin-mediated cell adhesion, cell-cell junction formation, cell proliferation and differentiation, cell survival, and neuronal signaling (Bos 2006; Roscioni *et al.* 2008; Kumar *et al.* 2018).

RGL1: Ral guanine nucleotide dissociation stimulator-like 1 is a small GTPase functioning in signal transduction (Sood *et al.* 2000). The Rap1-Rgl-Ral signaling network plays important role in regulating neuroblast cortical polarity and spindle orientation (Carmena *et al.* 2011).

RASA3: Ras GTPase-activating protein 3, a member of the GAP1 subfamily, is an inositol 1,3,4,5-tetrakisphophate-binding protein (Schurmans *et al.* 2015). The protein enhances the weak intrinsic GTPase activity of RAS proteins resulting in the inactive GDP-bound form of RAS (Cullen *et al.* 1995). It plays important roles in erythropoiesis, megakaryopoiesis, megakaryocyte adhesion and migration as well as integrin signaling (Blanc *et al.* 2012)

INPP5A: The protein encoded by this gene is inositol polyphosphate 5-phosphatase A, a membrane-associated enzyme. It hydrolyzes inositol polyphosphate, which regulates calcium release from intracellular stores and acts as a second messenger regulating cell proliferation and survival. Deletion of INPP5A causes progressive and permanent loss of cerebellar Purkinje cells in mice, suggesting its crucial role in Purkinje cell survival (Ooms *et al.* 2009; Yang *et al.* 2015).

IRAK4: Interleukin-1 receptor-associated kinase 4 is one of the four members of the IRAK family that plays an important role in signaling innate immune responses from Toll-like receptors. Accumulated evidence indicates its abnormal expression in inflammatory autoimmune disorders (Su *et al.* 2020). Interestingly, targeted degradation of IRAK4 was used for the treatment of cancer, neurodegenerative and cardiovascular diseases (Kargbo 2019a; Kargbo 2019b).

### Additional genes

ASB7: Ankyrin repeat and SOCS box protein 7 is an E3 ubiquitin ligase. It plays a crucial role in regulating spindle dynamics and genome integrity through targeting DDA3 for proteasomal degradation (Uematsu *et al.* 2016). A recent study also indicated the expression of ASB7 was elevated upon activation of the unfolded protein response pathway under endoplasmic reticulum stress (Anasa *et al.* 2018).

DENND11: DENN domain containing 11 protein is highly conserved in a wide range of animal species. Studies indicated its important role in neurogenesis and neuronal recovery in the hippocampus following transient cerebral ischemia (Zhang *et al.* 2007).

NKAPD1: NKAPD1 encodes an uncharacterized protein C11ORF57, which is also called NKAP Domain Containing 1.

SNTG1: SNTG1 encodes gamma-1 syntrophin, a neuronal cell specific protein. Syntrophins are scaffold cytoplasmic membrane proteins that bind signaling molecules, such as gamma-enolase for its neurotrophic activity (Hogan *et al.* 2001; Hafner *et al.* 2010). Gamma-1 synthrophin associates directly with dystrophin, a protein involved in the Duchenne muscular dystrophy (Bashiardes *et al.* 2004).

SS18: SS18 gene encodes a protein called synovial sarcoma translocated to X chromosome. Disruption of SS18 gene in mouse results in embryonic death due to placental failure. Fusion SS18-SSX1 is believed to underlie the pathogenesis of synovial sarcoma through elevated expression of the key Wnt target AXIN2 (De Bruijn *et al.* 2006; Cironi *et al.* 2016).

TEX11: TEX11, a male germ cell specific X-linked gene, encodes a testis expressed sequence 11 protein, which is essential for meiosis and male fertility in animals (Yatsenko *et al.* 2015).

UGP2: UGP2 gene encodes UTP-glucose-1-phosphate uridylyltransferase, an enzyme conserved from bacteria to human as a key player in glycogenesis and cell wall synthesis. In yeast, its expression contributes to oxidative stress response and long-term cell survival through production of storage carbohydrates. In higher animals, it is highly active in the liver and muscles (Smith and Rutter 2007; Yi and Huh 2015).

ZMYND12: Zinc finger MYND domain-containing protein 12 is conserved among higher eukaryotes. Its function is unknown.

In summary, we screened a previously established yeast model of FUS against a new collection of 13570 human genes and identified 37 suppressor genes of FUS induced toxicity in yeast. Although the identified suppressors have a wide range of cellular functions, genes encoding proteins involved in RNA and DNA binding are significantly overrepresented, suggesting a strong relationship between the toxicity of FUS in yeast and dysregulation in RNA metabolism, which is a widely considered major contributor to ALS pathogenesis (Polymenidou *et al.* 2012; Butti and Patten 2018).
